# DLPFC volume is a neural correlate of resilience in healthy high-risk individuals with both childhood maltreatment and familial risk for depression

**DOI:** 10.1017/S0033291721001094

**Published:** 2022-12

**Authors:** Katharina Brosch, Frederike Stein, Tina Meller, Simon Schmitt, Dilara Yuksel, Kai Gustav Ringwald, Julia-Katharina Pfarr, Lena Waltemate, Hannah Lemke, Nils Opel, Susanne Meinert, Katharina Dohm, Dominik Grotegerd, Janik Goltermann, Jonathan Repple, Alexandra Winter, Andreas Jansen, Udo Dannlowski, Igor Nenadić, Tilo Kircher, Axel Krug

**Affiliations:** 1 Department of Psychiatry and Psychotherapy, Philipps-Universität Marburg and University Hospital Marburg, UKGM, Rudolf-Bultmann-Str. 8, 35039 Marburg, Germany; 2 Center for Mind, Brain and Behavior (CMBB), Hans-Meerwein-Str. 6, 35032 Marburg, Germany; 3 SRI International, Center for Health Sciences, Bioscience Division, 333 Ravenswood Avenue, 94025 Menlo Park, CA, USA; 4 Department of Psychiatry, Westfälische Wilhelms-Universität Münster, Albert-Schweitzer-Campus 1, Building A9, 48149 Münster, Germany; 5 Core-Facility BrainImaging, Faculty of Medicine, Philipps-Universität Marburg, Rudolf-Bultmann-Str. 8, 35039 Marburg, Germany; 6 Department of Psychiatry and Psychotherapy, University of Bonn, Bonn, Germany

**Keywords:** Childhood maltreatment, DLPFC, resilience, risk factors, voxel-based morphometry

## Abstract

**Background:**

Two prominent risk factors for major depressive disorder (MDD) are childhood maltreatment (CM) and familial risk for MDD. Despite having these risk factors, there are individuals who maintain mental health, i.e. are resilient, whereas others develop MDD. It is unclear which brain morphological alterations are associated with this kind of resilience. Interaction analyses of risk and diagnosis status are needed that can account for complex adaptation processes, to identify neural correlates of resilience.

**Methods:**

We analyzed brain structural data (3T magnetic resonance imaging) by means of voxel-based morphometry (CAT12 toolbox), using a 2 × 2 design, comparing four groups (*N* = 804) that differed in diagnosis (healthy *v.* MDD) and risk profiles (low-risk, i.e. absence of CM and familial risk *v.* high-risk, i.e. presence of both CM and familial risk). Using regions of interest (ROIs) from the literature, we conducted an interaction analysis of risk and diagnosis status.

**Results:**

Volume in the left middle frontal gyrus (MFG), part of the dorsolateral prefrontal cortex (DLPFC), was significantly higher in healthy high-risk individuals. There were no significant results for the bilateral superior frontal gyri, frontal poles, pars orbitalis of the inferior frontal gyri, and the right MFG.

**Conclusions:**

The healthy high-risk group had significantly higher volumes in the left DLPFC compared to all other groups. The DLPFC is implicated in cognitive and emotional processes, and higher volume in this area might aid high-risk individuals in adaptive coping in order to maintain mental health. This increased volume might therefore constitute a neural correlate of resilience to MDD in high risk.

## Introduction

The risk for developing major depressive disorder (MDD) is increased when familial risk (i.e. having a first-degree relative with a psychiatric disorder) or childhood maltreatment (CM) are present in a person (Nanni, Uher, & Danese, 2012; Rasic, Hajek, Alda, & Uher, 2014). Yet, both risk factors only explain a small proportion of the variance in actual symptom presentation (Colodro-Conde et al., 2018; Lieb, Isensee, Höfler, Pfister, & Wittchen, 2003; Nanni et al., 2012). Besides risk factors that increase the risk for MDD, other factors must be at play that simultaneously mitigate these risks.

Familial risk increases the risk of developing the disorder from which the parent suffered (risk ratio, RR = 3.59), but also the risk for other psychiatric disorders (RR = 1.92) (Rasic et al., 2014). It is regarded as a proxy for genetic liability, but could also lead to a more stressful environment (Flory, Yehuda, Passarelli, & Siever, 2012). Healthy relatives with familial risk for MDD show similar gray matter volume (GMV) alterations as MDD patients, specifically reductions in the insula and orbitofrontal cortex (OFC) (Opel et al., 2016).

The environmental risk factor CM is associated with increased risk for developing depression (odds ratio, OR = 2.66–3.73), and within patients, CM is associated with recurrent or persistent depressive episodes (OR = 2.27) (Nanni et al., 2012; Nelson, Klumparendt, Doebler, & Ehring, 2017; Teicher & Samson, 2013). It is associated with increased social problems, negative cognitive bias, increased limbic responsiveness to negative stimuli, alterations in brain connectivity patterns, and persistent pro-inflammatory states of the immune system, among others (Baumeister, Akhtar, Ciufolini, Pariante, & Mondelli, 2016; Dannlowski et al., 2013; Günther, Dannlowski, Kersting, & Suslow, 2015; Meinert et al., 2019; Opel et al., 2019; Ouellet-Morin et al., 2011; Redlich et al., 2018). In healthy participants with CM, GMV changes were reported in the hippocampus, corpus callosum, anterior cingulate, OFC, and dorsolateral prefrontal cortex (DLPFC) (Dannlowski et al., 2016, 2012; Teicher, Samson, Anderson, & Ohashi, 2016).

Although there is a great focus in research on CM, few studies have investigated the combined effects of both CM and familial risk. The literature suggests that the presence of both risk factors in the same individual is associated with even worse mental health outcomes: in adolescents, this twofold risk was found to increase risk for several psychiatric disorders (MDD, autism spectrum disorders, conduct disorder, and generalized anxiety disorder) as well as to increase risk to attempt suicide, and increase risk of suicidal behavior up to OR = 4.43 (Greger, Myhre, Lydersen, & Jozefiak, 2015; Zelazny et al., 2019). In a study by Flory et al. (2012), such participants had an almost sixfold risk to develop post-traumatic stress disorder (PTSD) (OR = 5.89). A decreased volume in the bilateral hippocampal heads, in the DLPFC, medial prefrontal cortex (MPFC) and anterior cingulate cortex was reported in healthy participants with familial risk for MDD and history of emotional abuse (a subscale of the childhood trauma questionnaire) (Carballedo et al., 2012).

The risk factors CM and familial risk entail grave, adverse effects for the individual and are associated with morphometric changes already present in healthy individuals. However, the data clearly show that even the presence of both risks does not inevitably lead to the development of MDD. Clearly, such healthy high-risk individuals must be equipped with resources, positive coping skills, or traits which enable them to maintain mental health. Although many studies focus on the negative impact of risk factors, few studies investigate how some individuals at high risk still manage to maintain mental health, i.e. why they are resilient.

Resilience is a protective mechanism which is defined as the dynamic process to successfully adapt and cope with trauma, adversity, and negative stressors (Newman, 2005; Windle, 2011). Resilient individuals cope better with stress, report more positive emotions and are less likely to report depressive symptoms (Klasen et al., 2015; Ong, Bergeman, Bisconti, & Wallace, 2006). The concept of resilience encompasses both individual traits and skills, but also favorable environment, such as social support, intelligence quotient (IQ), and socioeconomic status (Ettner, 1996; Ozbay, Fitterling, Charney, & Southwick, 2008). The operationalization of resilience is a topic of fierce discussion, and operationalization in different studies varies greatly (Chmitorz et al., 2018; Kalisch, Müller, & Tüscher, 2015; Luthar, Cicchetti, & Becker, 2000). Some studies identify resilient individuals as those who do not develop PTSD after a traumatic event, which can only represent resilience to extreme events (Yehuda, Flory, Southwick, & Charney, 2006). Some rely on self-report questionnaires to assess resilience, which is termed trait resilience (Kahl, Wagner, de la Cruz, Köhler, & Schultz, 2018; Kalisch et al., 2019; Pangallo, Zibarras, Lewis, & Flaxman, 2015). These questionnaires measure skills such as cognitive reframing, ability to bounce back, acceptance, and personal competence, which have all been identified as skills relevant to resilience (Smith et al., 2008; Wagnild & Young, 1993). Direct stimulation of the left DLPFC (lDLPFC) and OFC using trans-cranial direct current stimulation (tDCS), increased levels of trait resilience as measured with a resilience questionnaire (Salehinejad, Nejati, & Derakhshan, 2017). Using questionnaires to assess resilience has shortcomings: first, self-reported resilience does not necessarily portray objective resilience, and second, without assessing adversity, questionnaires cannot represent the entire, process-like concept of resilience.

Other studies identify healthy, but at-risk individuals (e.g. with CM) as resilient. Resilience to CM has been associated with changes in the MPFC, and functional alterations in limbic areas such as hippocampus and amygdala (Ioannidis, Askelund, Kievit, & Van Harmelen, 2020; Moreno-López et al., 2020). High-risk but healthy adolescents were shown to have higher GMV in the right middle and superior frontal gyri (Burt et al., 2016). In a longitudinal investigation, it was found that those individuals who remained healthy despite high-risk were better able to access prefrontal regions for emotion regulation (Rodman, Jenness, Weissman, Pine, & McLaughlin, 2019).

Although results are mixed, it seems that the broader construct of resilience is associated with alterations in (pre)frontal regions that aid in planning, appraisal, executive functioning, and emotion regulation. Neuroimaging studies that identify healthy high-risk individuals as resilient have methodological shortcomings. In these, correlates of resilience are identified by either comparing healthy low-risk to healthy high-risk participants, or by comparing healthy high-risk to depressed high-risk participants. Although it might be possible to speculate about ‘neural resilience correlates’ using these comparisons, the former approach can *de facto* only identify the effects of risk status, while the latter can only identify the effects of diagnosis status (Amico et al., 2011).

Indeed, the interactive effect of risk and protective factors was reported in two studies at a phenotypical level, demonstrating risk and resilience contributed toward the likelihood of manifestation of MDD symptoms in an opposing, yet interactive manner (Navrady, Adams, Chan, Ritchie, & McIntosh, 2017; Wingo et al., 2010). These findings highlight that it is not the mere absence of risk factors that help maintain mental health but also the presence of resilience factors, and that the relationship between risk and resilience is complex and interactive.

In summary, (1) there is a lack of studies that investigate the subgroup of healthy high-risk individuals with both CM and familial risk, (2) other studies investigating risk factors have methodological shortcomings in the operationalization of resilience, and (3) only the interaction of risk × diagnosis can give information about resilience-specific morphometric alterations.

In the current study, high risk is operationalized as the presence of the two risk factors CM and familial risk. Low risk is operationalized as the absence of both CM and familial risk. Resilience is operationalized as maintaining mental health despite the presence of the two risk factors CM and familial risk. In our study, we aim to examine brain morphometric correlates of resilience of healthy, high-risk participants, compared to healthy low-risk participants, depressed high-risk participants, and depressed low-risk participants. We employ a 2 × 2 group design: risk (high risk *v.* low risk) × diagnosis: (healthy *v.* depressed), investigating the interaction effect. Based on the literature, we expect to find higher volume in frontal regions which might counteract the adverse effects of risk and aid in the maintenance of mental health.

## Methods and materials

### Sample

For the current study, we selected a sample of *N* = 804 subjects of the FOR2107 cohort (Marburg-Münster Affective Disorder Cohort Study) study (http://www.for2107.de). FOR2107 is an ongoing longitudinal, bicentric cohort study investigating the neurobiology of affective [major depression and bipolar disorder (BP)] and psychotic disorders [schizophrenia (SZ) and schizoaffective disorder]. Participants are deeply phenotyped using questionnaires, neuropsychological testing, neuroimaging (functional and structural), and collection of biomaterial (for a detailed description see Kircher et al., 2018). Data are collected at baseline, and 2 and 5 years afterward. Data for this study were collected between 2014 and 2018, with a median of data collected in 2016. Healthy participants, and patients (both acute and remitted) were recruited via newspaper advertisements; patients were additionally recruited via local in- and outpatient services in Marburg and Münster, Germany. Written informed consent was obtained from all subjects before participation, and participants received a financial compensation afterward. Participants with a history of substance dependence, neurological disorders, severe medical disorders, head trauma, or IQ <80 were excluded. We included participants aged 18–65. Healthy participants with no history of mental illness were included. The study was approved by the ethics committees of the respective recruitment sites in accordance with the Declaration of Helsinki. The German version of the structured clinical interview for DSM-IV-TR (SCID-I) (Wittchen, Gruschwitz, Wunderlich, & Zaudig, 1997) was used to diagnose participants by trained raters.

We employed a 2 × 2 (diagnosis × risk) design, distinguishing healthy participants (HC) and depressed patients (MDD) (factor diagnosis) at either low risk (R−) or high risk for MDD (R+) (factor risk). R− reported neither familial risk nor CM, while R+ reported both familial risk and CM. The descriptive data of the sample can be found in Table 1.

**Table 1.** Descriptive sample statistics

	HCR− (*n* = 437)	HCR+ (*n* = 67)	MDDR− (*n* = 101)	MDDR+ (*n* = 199)	*p*
Age	32.86 (12.56)	35.91 (14.49)	35.04 (13.08)	36.32 (12.70)	0.008*
Sex, *n*, female (male)	269 (168)	52 (15)	55 (46)	140 (59)	0.003*
HAM-D	1.05 (1.82)	2.10 (2.81)	6.81 (5.60)	8.68 (6.49)	<0.001*^a^
BDI	3.30 (3.63)	5.73 (4.87)	13.93 (10.17)	18.03 (10.87)	<0.001*^a^
Total duration of illness, months	NA	NA	20.10 (32.84)	49.80 (77.74)	<0.001*
Medication type, *n*					
SSRI	NA	NA	24	50	0.888
SNRI	NA	NA	20	46	0.558
NaSSA	NA	NA	10	11	0.230
Antipsychotic	NA	NA	23	29	0.106

*The significant of bold values indicated p values < 0.05.

The mean values are reported, with the standard deviation in parentheses, unless otherwise specified. Bonferroni post-hoc tests were used to compare the groups.

HCR−, healthy control and low risk; HCR+, healthy control and high risk; MDDR−, depressive and low risk; MDDR+, depressive and high risk; HAM-D, Hamilton Depression Rating Scale, BDI, Beck Depression Inventory.

a HC groups differ significantly from MDD groups, MDD groups differ significantly from each other: HCR− < MDDR−; HCR− < MDDR+; HCR+ < MDDR−; HCR+ < MDDR+; MDDR− < MDDR+.

### Assessment of familial risk

Familial risk was defined as having a first-degree relative with MDD, BP, or SZ, and assessed as part of a questionnaire battery, asking if the participant had a parent, sibling, or child who had ever been treated for one of these disorders. In the R− group, only participants not reporting any psychiatric problems in any first-degree relatives were included.

### Assessment of CM

The German version of the Childhood Trauma Questionnaire (CTQ) was used to assess CM retrospectively (Wingenfeld et al., 2010). This self-report questionnaire consists of five subscales: emotional abuse, physical abuse, sexual abuse, emotional neglect, physical neglect; with five items per scale rated on a 5-point Likert scale. A higher score indicates a higher degree of maltreatment. Individuals were classified as having experienced CM when the respective cut-off score according to Walker et al. (1999) was met in at least one of the five scales.

### Magnetic resonance imaging (MRI) data acquisition

At both sites, high-resolution T1 images were acquired with a 3T whole body MRI scanner (Marburg: 12-channel head matrix Rx-coil, Tim Trio, Siemens, Erlangen, Germany; Münster: 20-channel head matrix Rx-coil, Prisma, Siemens, Erlangen, Germany). A 3D fast gradient echo sequence (MPRAGE) was used with the following configurations: Marburg: field of view (FOV) = 256 mm, 176 slices, repetition time (TR) = 1900 ms, echo time (TE) = 2.26 ms, inversion time (TI) = 900 ms, slice thickness = 1 mm, voxel size = 1 × 1 × 1 mm, flip angle = 9° and Münster: FOV = 256 mm, 192 slices, TR = 2130 ms, TE = 2.28 ms, TI = 900 ms, slice thickness = 1 mm, voxel size = 1 × 1 × 1 mm, flip angle 8°. Image quality was assessed using the quality assurance protocols of the CAT12 toolbox.

The CAT12 toolbox (build 1184, Gaser, Structural Brain Mapping group, Jena University Hospital, Jena, Germany) implemented in SPM12 (v7771, Statistical Parametric Mapping, Institute of Neurology, London, UK), running under MATLAB (version v2017a, The MathWorks, USA) was used for pre-processing of scans using default parameter settings. Images were segmented into gray matter, white matter, and cerebrospinal fluid and spatially normalized using the DARTEL algorithm. All images passed individual quality control, checking for artifacts and image quality. Data were smoothed with an 8 mm full-width half-maximum Gaussian kernel, using an absolute threshold of 0.1.

### Statistical analyses

Data were analyzed using SPSS25 and SPM12. We conducted an analysis of covariance (ANCOVA) interaction analysis using a 2 × 2 design in SPM, including the covariates age, sex, total intracranial volume, as well as body coil and site, in accordance with our MRI quality assurance protocol (Vogelbacher et al., 2018). We examined four bilateral ROIs and applied small volume correction using family-wise error (FWE) correction. ROIs were defined using the Neuromorphometrics atlas implemented in SPM12.

To examine the interaction effect of significant ROIs, we extracted eigenvariate values, which are an approximation of mean volume inside the cluster and compared them in SPSS using ANCOVA (correcting for all covariates).

### ROI selection

Drawing on the literature and our hypotheses, we selected four bilateral ROIs that had previously been implicated in both resilience research and the investigation of healthy high-risk participants (CM and/or familial risk) for the interaction analysis. Overlapping areas in both risk and resilience research are (pre)frontal areas. We therefore selected four bilateral ROIs using the Neuromorphometrics atlas: *middle frontal gyrus* (MFG) (Burt et al., 2016; Carballedo et al., 2012; Salehinejad et al., 2017), *superior frontal gyrus* (Burt et al., 2016), *inferior frontal gyrus* (*orbital part*) (Opel et al., 2016), and *frontal pole* (Carballedo et al., 2012; Moreno-López et al., 2020; Teicher et al., 2016).

## Results

**Fig. 1.**
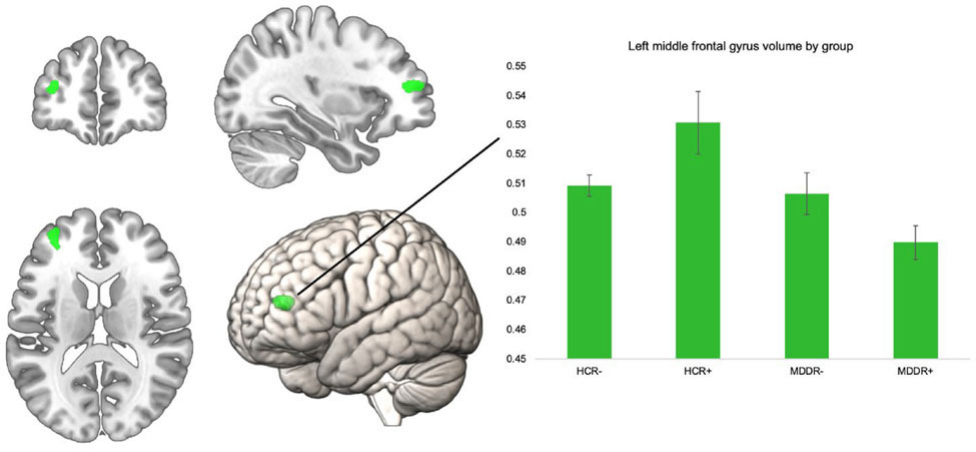
Significant left MFG ROI for interaction of risk × diagnosis, volume in the left MFG by group. Error bars indicate 1 standard error of the mean.

To investigate the interactive effect of diagnosis and risk on GMV, we performed an interaction analysis using the four bilateral ROIs. The interaction analysis did not reveal significant findings for the bilateral superior frontal gyri, the frontal poles, pars orbitalis of the inferior frontal gyrus, and the right MFG, *p* > 0.05. There was a significant effect for the left MFG ROI at peak level (*k* = 199, *x*/*y*/*z* = −34/48/12, *T* = 4.06, *p* = 0.047, FWE corrected). To examine this interaction effect by group, we extracted eigenvariate values of this cluster and compared them in SPSS using an ANCOVA (correcting for all covariates) (Fig. 1).

The groups differed significantly in their left MFG volume *F*_(3,796)_ = 10.32, *p* < 0.001, *η*^2^ = 0.037. We compared HCR+ to the other three groups using Bonferroni post-hoc comparisons. HCR+ had significantly higher volumes compared to all other groups. HCR+ *v*. HCR−; HCR+ *v*. MDDR−, and HCR+ *v*. MDDR+: all *p* < 0.001. The ROI and the mean volumes per group are shown below. Including body mass index or years of education as an additional covariate in the model did not alter the results, nor did excluding obvious GMV outliers (see online Supplementary Table 2). To further investigate robustness of our finding, we analyzed this interaction effect in only women (online Supplementary Table 2), and in an additional sample of healthy controls with intermediate risk (online Supplementary Table 3). The supplement also lists the results of the whole-brain analysis (online Supplementary Table 4).

## Discussion

Our results reveal higher volume in the left MFG, part of the DLFPC, in healthy participants with two risk factors (CM and familial risk) for MDD, compared to depressed patients irrespective of risk status, and compared to healthy low-risk individuals. To our knowledge, this is the first study investigating GMV in healthy high-risk (both CM and familial risk) in a 2 × 2 design. The DLFPC is associated with cognitive flexibility, reappraisal, and impulse control (Steinbeis, Bernhardt, & Singer, 2012). These skills are known to contribute to mental health and are impaired in MDD. Increased volume in this region could mitigate risk effects in already at-risk individuals. Skills such as reframing and cognitive flexibility have already been identified as parts of resilience (Iacoviello & Charney, 2014). The DLPFC might not only be implicated in cognitive processes, but also in emotional ones: administration of repetitive transcranial magnetic stimulation to the left DLFPC in MDD patients increased empathetic happiness and decreased anhedonia (Light, Bieliauskas, & Taylor, 2019). Using tDCS of the lDLPFC combined with gaze-contingent training was shown to improve attention regulation in low-resilient individuals (Sanchez-Lopez et al., 2020). DLPFC volume was furthermore shown to be a mediator between socioeconomic status and executive functioning (Shaked et al., 2018). This also highlights the importance of environmental factors important in the maintenance of mental health (Ettner, 1996; Ozbay et al., 2008). Our results point to the lDLPFC as a neural correlate of resilience in high-risk individuals. Higher volume in the DLPFC might aid these at-risk individuals both on a cognitive- and on an emotional level in maintaining mental health.

It is important to note that healthy, high-risk individuals with both CM and familial risk constitute a unique subgroup of healthy participants. Drawing from a sample of *n* = 1500 healthy individuals, only *n* = 67 fit the criteria for HCR+, while *n* = 437 healthy individuals reported no such risk factor. On the other hand, risk distribution in MDD was remarkably different; here, *n* = 199 depressive participants fit the same risk criteria (χ^2^(1, *N* = 804) = 238.97, *p* < 0.001). This shows that our HCR+ group constitutes a rare, highly resilient subgroup that can only be investigated in a large sample of participants. Our results point to the lDLFPC as a protective morphometric correlate of resilience that aids participants with both CM and familial risk in the maintenance of mental health.

### Limitations

We limited detection to previously selected ROIs. These ROIs were based on solid previous research and enabled us to purposefully identify regions associated with resilience. Our groups cannot represent the heterogeneity of MDD patients or risk groups, as we excluded participants with only CM or familial risk present. However, they do provide valuable insight into the understanding of high- and low-risk groups. Environmental risk was assessed using the CTQ questionnaire, which – as a self-report instrument – has strengths and limitations (Baldwin, Reuben, Newbury, & Danese, 2019; Goltermann, Opel, & Dannlowski, 2019; MacDonald et al., 2016). Participants were identified as having CM when they exceeded at least one of the five subscales of the CTQ. This approach can be problematic, and a continuous assessment of maltreatment (using the sum score) would be valuable, however, this was not feasible using this model. Our groups can still separate between high- and low-risk, as low-risk groups did not have either familial risk, nor exceed one scale of the CTQ. We included both remitted and acute MDD patients, which might have made MDD groups more heterogenous. Familial risk was defined as having a first-degree relative with MDD, BP, or SZ. Distinct morphometric changes have been described for the relatives of MDD and SZ patients, but linkage and recent genome-wide association studies have emphasized the substantial genetic overlap among BP, SZ, and MDD (Anttila et al., 2018; McDonald et al., 2004; Smoller et al., 2019). We operationalized resilience as the outcome of mental health, despite the presence of CM and familial risk. Trait questionnaires would be able to assess self-reported resilience skills such as reframing and optimism. However, mental health despite high risk is a much more objective and direct measure of resilience than subjective, self-reported resilience.

### Future directions

Our study identifies morphometric correlates of resilience in a highly resilient subgroup of individuals at high risk (both CM and familial risk) to develop MDD. This group should be examined in more detail in future research. In our study, HCR+ did not show uniquely higher scores in other relevant measures such as IQ, income, education, or trait resilience (see online Supplementary Table 1). Studies investigating the effects of MDD and other psychiatric disorders should take into account the effects of risk and resilience, as they both contribute in a unique way to either increase or decrease the risk for these disorders. The concrete cognitive and behavioral correlates of higher volume in the lDLPFC, which might be improved emotional attentional control and cognitive flexibility, among others, should be investigated in future studies.

Bolstering resilience by teaching such techniques should be integrated as a central goal in both prevention and psychotherapy. Resilience seems to be the default response to adversity, rather than the exception. Considering the grave impact of risk factors, this adaptive coping equals an ‘ordinary magic’, and resilience factors both on a morphometric, and a behavioral level deserve more attention in future research (Masten, 2001).

## Supplementary material

For supplementary material accompanying this paper visit https://doi.org/10.1017/S0033291721001094. click here to view supplementary material
