# Supplementary material for: DLPFC volume is a neural correlate of resilience in healthy high-risk individuals with both childhood maltreatment and familial risk for depression
Source: Psychol Med. 2021 Apr 16;52(16):4139–45. doi: 10.1017/S0033291721001094 (PMC9811272; doi:10.1017/S0033291721001094)
Supplement: Supplementary file 1 [file S0033291721001094sup001.docx]

**Supplementary Material**

Brosch et al., DLPFC volume is a neural correlate of resilience in healthy high-risk individuals with both childhood maltreatment and familial risk for depression

1. **Investigation of further covariates and resilience factors**

Table 1. Additional state/trait scores by group

|  | **HCR-**  (n=437) | **HCR+**  (n=67) | **MDDR-**  (n=101) | **MDDR+**  (n=199) | *p* |
| --- | --- | --- | --- | --- | --- |
| BMI | 24.07  (4.60) | 23.45 (3.40) | 25.84  (5.29) | 26.79  (6.33) | <. 001 ^a)^ |
| Years of education | 14.22  (2.54) | 14.17  (2.46) | 13.52  (2.66) | 13.16  (2.83) | <. 001 ^b)^ |
| RS-25  Sum | 143.69 (16.70) | 139.58 (19.17) | 119.97 (25.95) | 111.86 (25.90) | <. 001 ^c)^ |
| RS-25  Acceptance of self and life | 44.14  (6.16) | 41.37  (7.49) | 35.47  (9.60) | 31.95  (9.11) | <. 001 ^d)^ |
| RS-25  Personal competence | 99.55  (11.71) | 98.21  (12.85) | 84.50  (17.73) | 79.91  (18.45) | <. 001 ^c)^ |
| FSozu | 4.61  (0.44) | 4.37  (0.55) | 4.15  (0.73) | 3.75  (0.90) | <. 001 ^d)^ |
| Household net income (€) | 2025.10  (1897.35) | 2162.51  (2031.83) | 1835.07  (1558.22) | 1706.59  (1332.36) | .128 |
| IQ | 115.18  (13.26) | 114.24  (12.64) | 110.89  (12.39) | 112.80  (13.61) | .015 ^e)^ |

*Note:* Mean (*SD*). Bonferroni post-hoc tests were used to compare the groups. HCR- : Healthy control and low risk, HCR+: healthy control and high risk, MDDR-: depressive and low risk, MDDR+: depressive and high-risk. RS-25 = resilience questionnaire, FSozu = social support,IQ measured using Multiple Choice Word Test-B (MWT-B)

1. HC groups differ significantly from MDD groups:

HCR- < MDDR-; HCR- < MDDR+; HCR+ < MDDR-; HCR+ < MDDR+

1. HC groups differ significantly from MDDR+ : HCR - > MDDR+; HCR+ > MDDR+
2. HC groups differ significantly from MDD groups, MDD groups differ significantly from each other: HCR- > MDDR-; HCR- > MDDR+; HCR+ > MDDR-; HCR+ > MDDR+; MDDR- > MDDR+
3. All groups differ significantly from each other:

HCR- > HCR+; HCR- > MDDR-; HCR- > MDDR+; HCR+ > MDDR-; HCR+ > MDDR+; MDDR- > MDDR+

1. HCR+ differs significantly from MDDR- : HCR+ > MDDR
2. **Re-analysis of risk x diagnosis interaction effect with additional covariates (BMI, socioeconomic status), and in only women**

Table 2. Additional GMV analyses

|  |  | MNI (at peak) | | | |  |  |
| --- | --- | --- | --- | --- | --- | --- | --- |
|  | Cluster size: k | *x* | *y* | *z* | Side | T | *p*  (FWE corr.) |
| **Analysis with BMI as covariate** |  |  |  |  |  |  |  |
| Middle Frontal Gyrus | 186 | -34 | 48 | -12 | L | 4.06 | .046 |
| Superior Frontal Gyrus | 55 | 26 | 20 | 63 | R | 3.34 | .401 |
| **Analysis with years of education as covariate** |  |  |  |  |  |  |  |
| Middle Frontal Gyrus | 206 | -34 | 48 | 12 | L | 4.08 | .038 |
| Superior Frontal Gyrus | 10 | 26 | 20 | 63 | R | 3.21 | .478 |
| **Analysis with only women (N = 516)** |  |  |  |  |  |  |  |
| Middle Frontal Gyrus | 11 | -34 | 48 | 12 | L | 3.22 | .506 |

*Note:* Socioeconomic status was operationalized as years of education. Other covariates included in each model are age, sex, site, body coil change and TIV. We applied ROI analysis using four literature-derived bilateral ROIs (i.e. middle frontal gyrus, superior frontal gyrus, inferior frontal gyrus, frontal pole).

Group distribution in the female sample: HCR- (*n*=269), HCR+ (*n*=52), MDDR- (*n*=55), MDDR+ (*n*=140)

1. **Inclusion of HC with only environmental or familial risk**

Table 3. Additional analysis of healthy controls including intermediate risks

|  | **HCR-**  (n=437) | **HCenv**  (n=213) | **HCfam**  (n=127) | **HCR+**  (n=67) | *p* |
| --- | --- | --- | --- | --- | --- |
| lMFG | 0.503  (0.076) | 0.479 (0.080) | 0.516  (0.077) | 0.515  (0.086) | <. 001 ^a)^ |

Note: Intermediate risk = only one risk facter, i.e. environmental or maltreatment.

HCR- and HCR+ are groups previously defined, HCenv = healthy control with only environmental risk, HCfam = healthy control with only familial risk

We extracted average grey matter values and controlled for age, sex, site, body coil change and total intracranial volume. Due to novel calculations lMFG volume scores differ minimally in the HCR- and HCR+ group.

The groups differ significantly in their volume in the left middle frontal gyrus: *F*(3,835) = 7.374, *p* < .001. Bonferroni post-hoc revealed a significant difference between HCR+ and HCR-, and HCR+ and HCenv

^a)^ HCR- < HCR+; HCenv < HCR

1. **Investigation of possible GMV outliers**


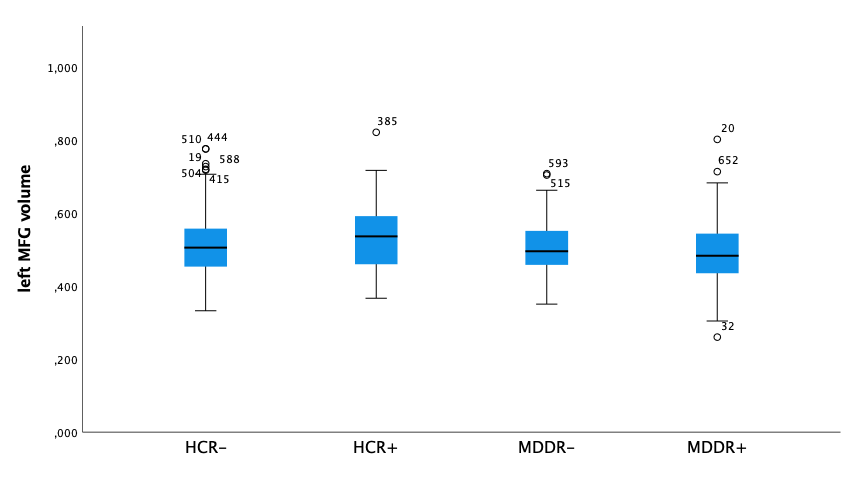


Figure 1. Boxplots for left MFG volume per group

*Note*: These values are not adjusted for relevant covariates (i.e. age, sex, site, body coil, TIV)


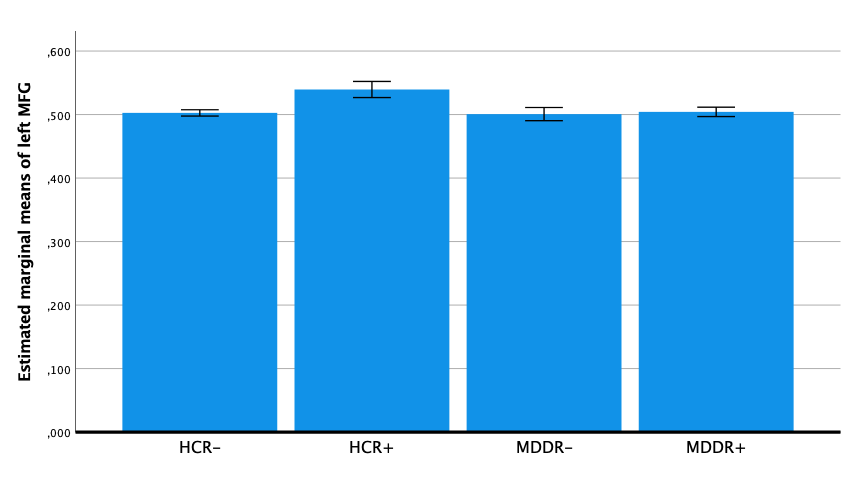


Figure 2. Estimated marginal means of left MFG volume by group adjusted for relevant covariates (i.e. age, sex, site, body coil, TIV).

Table 4: lMFG volume per group after exclusion of outliers

|  | Mean | N | Standard deviation | Minimum | Maximum |
| --- | --- | --- | --- | --- | --- |
| HCR- | .509 | 437 | .077 | .332 | .775 |
| HCR+ | .531 | 67 | .087 | .367 | .820 |
| MDDR- | .507 | 101 | .071 | .350 | .707 |
| MDDR+ | .490 | 199 | .082 | .260 | .801 |

Note: We excluded all outliers (*n* = 12) and re-ran the analysis. Groups differed significantly in their volumes *F*(3, 784) = 9.99, *p* < .001. HCR+ still had significantly higher lMFG volume compared to all other groups, Bonferroni post-hoc corrected, all *p* <.001.

1. **Exploratory whole-brain analysis risk x diagnosis interaction at *p* < .001, uncorrected**

|  |  | MNI (at peak) | | | |  |  |
| --- | --- | --- | --- | --- | --- | --- | --- |
|  | Cluster size: k | *x* | *y* | *z* | Side | T | *p*  (FWE corr. peak level) |
| **Positive contrast** |  |  |  |  |  |  |  |
| Fusiform Gyrus | 26 | 34 | -4 | -32 | R | 3.44 | .929 |
| **Negative contrast** |  |  |  |  |  |  |  |
| Middle Frontal Gyrus | 268 | -34 | 48 | 12 | L | 4.06 | .305 |
| Postcentral Gyrus | 56 | 30 | -30 | 66 | R | 3.42 | .940 |
| Basal Cerebrum and Forebrain Brain | 12 | 10 | 0 | -8 | R | 3.28 | .986 |
| Inferior Frontal Gyrus | 7 | 58 | 20 | 3 | R | 3.21 | .993 |
| Superior Frontal Gyrus | 7 | 26 | 20 | 63 | R | 3.20 | .995 |
| Angular Gyrus | 1 | -32 | -68 | 39 | L | 3.11 | .999 |
